# A Case of Ruptured Decidualized Ovarian Endometrioma: Usefulness of Serial MRI for Determining Adequate Management

**DOI:** 10.1155/2022/3234784

**Published:** 2022-07-31

**Authors:** Saki Yamamoto, Tomohiro Kikuchi, Hiroyuki Fujii, Yuko Otake, Mitsuru Matsuki, Risa Narumi, Masashi Endo, Hiroyuki Fujiwara, Harushi Mori

**Affiliations:** ^1^Department of Radiology, Jichi Medical University School of Medicine, Tochigi, Japan; ^2^Department of Obstetrics and Gynecology, Jichi Medical University School of Medicine, Tochigi, Japan

## Abstract

Decidualization can originate in ovarian endometrioma by elevated serum progesterone levels during pregnancy, which mimics malignancy on ultrasonography. Moreover, decidualized ovarian endometrioma may rupture and cause acute abdominal pain during pregnancy. Magnetic resonance imaging (MRI) is reportedly useful in differentiating decidualized ovarian endometriomas from malignancies. However, to our knowledge, serial MRI of decidualized ovarian endometrioma before and after rupture has not been reported. Herein, we report the case of a 39-year-old woman with a ruptured decidualized ovarian endometrioma in which serial MRI was useful for adequate management. She had a history of right ovarian endometrioma. Transvaginal ultrasonography at 20 weeks of gestation showed the known right ovarian endometrioma with mural nodules that were not evident before pregnancy. MRI for further evaluation showed ovarian endometrioma with mural nodules with signals similar to those of the placenta. Based on the MRI findings, we diagnosed a decidualized ovarian endometrioma. At 27 weeks of gestation, she complained of sudden abdominal pain, for which MRI was performed. MRI showed disappearance of the ovarian endometrioma and bloody ascites, based on which we diagnosed a ruptured ovarian endometrioma. The abdominal pain subsided immediately, and a conservative observational treatment approach was taken. At 37 weeks of gestation, right ovarian cystectomy was performed simultaneously with an elective cesarean section, which revealed a ruptured decidualized ovarian endometrioma. Our findings demonstrate that the accurate diagnosis of a ruptured decidualized ovarian endometrioma on serial MRI can contribute to its management.

## 1. Introduction

Ovarian endometrioma occurs in 17–44% of patients with endometriosis and accounts for 35% of all benign ovarian cysts [1]. During pregnancy, increased progesterone levels may cause the decidualization of ovarian endometriomas, which mimics malignancies on imaging [2]. Recently, magnetic resonance imaging (MRI) has been reported to be useful in differentiating decidualized tissue from malignancies [3]. Moreover, decidualization can increase the risk of rupture of ovarian endometrioma [4]. Therefore, accurate diagnosis of a ruptured decidualized ovarian endometrioma during pregnancy is important for its adequate management.

Herein, we report a case of ruptured decidualized ovarian endometrioma in which serial MRI findings before and after the rupture were useful for determining the appropriate management.

## 2. Case Presentation

**Figure 1:**
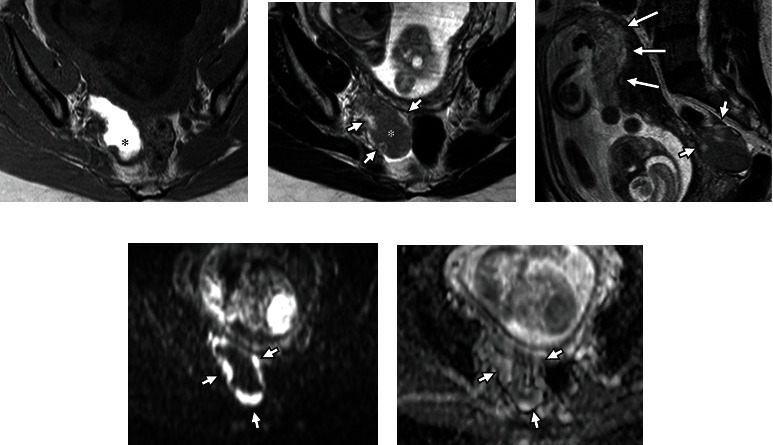
Magnetic resonance imaging (MRI) of the pelvis performed at 21 weeks of gestation. (a) Axial T1-weighted image. (b) Axial T2-weighted image. (c) Sagittal T2-weighted image. (d) Axial diffusion-weighted image (b factor: 1000 s/mm^2^). (e) Apparent diffusion coefficient (ADC) map. MRI shows a well-circumscribed teardrop-shaped cystic lesion with mural nodules measuring 68 × 45 × 35 mm in the right ovary. The cystic lesion shows uniformly marked high signal intensity on the T1-weighted image (a, asterisk) and intermediate signal intensity (b, asterisk) on T2-weighted images consistent with ovarian endometrioma. The mural nodules show intermediate to high signal intensity on T2-weighted images (b, c; short arrows) similar to the placenta (c, long arrows). Diffusion-weighted image (d, short arrows) and ADC map (e, short arrows) show the mural nodules with a high ADC value (1.6 × 10^−3^ mm^2^/s). Based on these imaging findings, we diagnosed a decidualized ovarian endometrioma.

A 39-year-old woman (gravida 0, para 0) presented to our hospital for infertility treatment. She had a history of a 30 mm diameter right ovarian endometrioma. She became pregnant by oral clomiphene-intrauterine artificial insemination. At 20 weeks of gestation, transvaginal ultrasonography revealed that the ovarian endometrioma had enlarged to 50 mm in diameter with mural nodules. The patient's serum cancer antigen-125 levels were slightly elevated (37 U/mL; normal, <35 U/mL), and carbohydrate antigen 19-9 levels were within normal limits (16 U/mL; normal, <37 U/mL). MRI for further evaluation showed a well-circumscribed, teardrop-shaped cystic lesion with mural nodules measuring 68 × 45 × 35 mm in the right ovary (Figure 1). The cystic lesion showed uniformly marked high signal intensity on T1-weighted images and intermediate signal intensity on T2-weighted images, consistent with an ovarian endometrioma. The mural nodules showed intermediate to high signal intensity on T2-weighted images, high signal intensity on diffusion-weighted images, and a high apparent diffusion coefficient (ADC) value (1.6 × 10^−3^ mm^2^/s) on the ADC map, which were similar to those of the placenta. Based on these imaging findings, we diagnosed a decidualized ovarian endometrioma. Further, we planned to perform MRI 6 weeks later.

**Figure 2:**
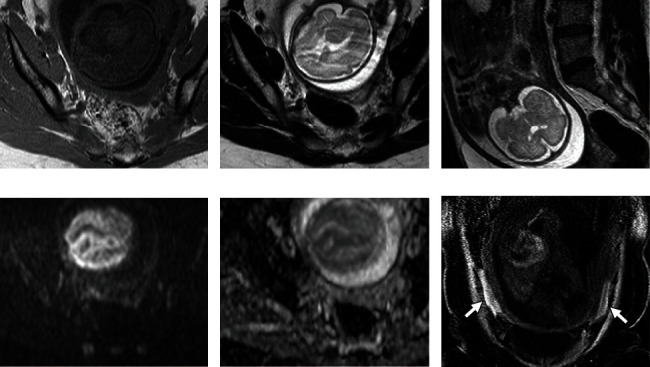
Magnetic resonance imaging (MRI) of the pelvis performed at 27 weeks of gestation. (a) Axial T1-weighted image. (b) Axial T2-weighted image. (c) Sagittal T2-weighted image. (d) Axial diffusion-weighted image (b factor: 1000 s/mm^2^). (e) ADC map. (f) Coronal T1-weighted image with fat saturation. The cystic lesion disappears (a–e). Ascites around the uterus shows high signal intensity on fat-suppressed T1-weighted images, which is consistent with bloody ascites (f, arrows). Based on serial MRI findings, rupture of the decidualized ovarian endometrioma is suspected.

At 27 weeks of gestation, the patient complained of sudden abdominal pain. Emergent MRI was performed. The decidualized ovarian endometrioma had disappeared; however, bloody ascites were found (Figure 2). Based on serial MRI findings, rupture of the decidualized ovarian endometrioma was suspected. The abdominal pain subsided immediately after injection of pentazocine 15 mg, and conservative observational management was chosen.

**Figure 3:**
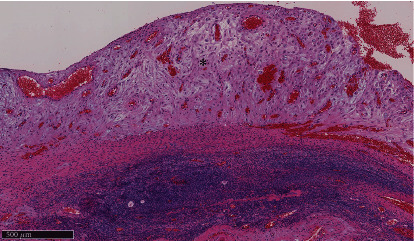
Microscopic view of a mural nodule (hematoxylin and eosin staining, magnification: 100x). Edematous tissue with abundant stromal cells' cytoplasm is visible (asterisk). These findings are consistent with decidualization.

At 37 weeks of gestation, the patient underwent an elective cesarean section and right ovarian cystectomy simultaneously. Ruptured ovarian cystic lesions were confirmed during the surgery. The cystic lesion contained chocolate-like bloody fluid. Histopathological findings revealed decidual reaction of the ovarian endometrioma and no evidence of malignancy (Figure 3). The final diagnosis was a ruptured decidualized ovarian endometrioma. The postoperative course was uneventful, and the patient was discharged 7 days later.

## 3. Discussion

We have reported the case of a ruptured decidualized endometrioma during pregnancy, which was useful to diagnose accurately by serial MRI and determine the patient's treatment options. In the present case, we diagnosed a decidualized ovarian endometrioma upon the first MRI examination during pregnancy. Further, we suspected rupture of the endometrioma, due to its disappearance and bloody ascites found on serial MRI at the onset of sudden abdominal pain. We further employed a conservative observational management approach, and elective cesarean section and right ovarian cystectomy were simultaneously performed at full term. Intraoperative and histologic examination demonstrated a ruptured decidualized endometrioma.

Endometriosis is a common disease occurring in 5–10% of women of reproductive age [2]. It is one of the main causes of infertility due to mechanical factors, including adhesions, tubal blockage, and ovulation disorders [5]. The number of pregnancies with endometriosis has been increasing due to the progression of assisted reproductive technologies [2]. Decidualization can also originate in ovarian endometrioma by elevating serum progesterone levels during pregnancy [2]. Previous reports revealed that approximately 12% of ovarian endometriomas have decidual reactions during pregnancy [2]. Decidualization primarily occurs in the second trimester and regresses during the third trimester or postpartum period [2].

Ovarian endometriomas can rupture for various reasons, including adhesions, tissue fragility, increased external pressure, or increased internal pressure due to mass enlargement. During pregnancy, increased levels of progesterone tend to shrink ovarian endometrioma [2]. However, decidualization is expected to increase ovarian endometrioma rupture due to enlargement and wall softening caused by a severe inflammatory response [2, 6]. To date, there have been six case reports of ruptured decidualized ovarian endometrioma (Table 1) [2, 7–11]. Among the six cases, rupture occurred during the first trimester in two cases, second trimester in three cases, and third trimester in one case.

Ultrasonography is the first choice for the screening of decidualized ovarian endometrioma. However, decidualized tissues appear as mural nodules in the cyst mimicking malignant tumors, such as endometrioid carcinoma and clear cell carcinoma arising from ovarian endometrioma [12]. Moreover, no previously reported cases (Table 1) were accurately diagnosed preoperatively with ruptured decidualized ovarian endometrioma. It is considered that ultrasonography may be limited of the visual field during pregnancy.

MRI is reported to be useful in the diagnosis of decidualized tissue, which has a thin broad structure and shows intermediate to high signal intensities on T2-weighted images, similar to the placenta. Morisawa et al. reported that the ADC values of the decidualized regions were significantly higher than those of the mural nodules of endometrioid carcinoma or clear cell carcinoma (1.77 × 10^−3^ mm^2^/s vs. 1.13 × 10^−3^ mm^2^/s) on diffusion-weighted images [3]. In the present case, the patient's MRI findings were similar, and we diagnosed a decidualized ovarian endometrioma. Additionally, MRI of a ruptured endometrioma can identify shrinkage or disappearance of the cyst and bloody ascites, as in the present case [13].

To our knowledge, serial MRI before and after the rupture of decidualized ovarian endometrioma during pregnancy has not been reported. In the present case, serial MRI findings before and after rupture showed disappearance of the cyst with MRI findings characteristic of decidualized ovarian endometrioma, which contributed to the accurate diagnosis.

There is no definite management for ruptured benign ovarian cystic lesions during pregnancy. All of the six patients mentioned in Table 1 underwent surgery. However, the risk of miscarriage associated with abdominal surgery during pregnancy increases in the first trimester. In addition, the risk of adverse outcomes such as miscarriage, preterm delivery, and intrauterine fetal death is increased in surgeries performed after 23 weeks of gestation [14]. Therefore, we believe that conservative treatment can be a management option in cases without torsion or malignancy and with stable hemodynamics or pain.

In conclusion, here we accurately diagnosed a ruptured decidualized ovarian endometrioma by serial MRI and chose conservative observation. In the future, the indications for conservative treatment of benign ovarian cystic lesions during pregnancy should be considered with the addition of MRI findings.

## Figures and Tables

**Table 1:** Summary of seven cases of ruptured decidualized endometriotic cysts.

Authors (year)	Patient age (years)	Size (mm)	Time of rupture	Imaging	Preoperative diagnosis	Treatment during pregnancy
Vercellini et al. (1992) [11]	29	80	Third trimester (35 weeks pregnant)	US	Bowel obstruction	Laparotomy (cyst enucleation and a cesarean section)
Loh et al. (1998) [9]	25	40	First trimester (6 weeks pregnant)	US	Ectopic pregnancy	Laparoscopic ovarian cystectomy
Garcia-Velasco et al. (1998) [7]	25	83 × 54	First trimester (9 weeks pregnant)	US	Hemorrhagic corpus luteum or endometrioma	Laparotomy (left salpingo-oophorectomy)
Gregora et al. (1998) [8]	44	60	Second trimester (17 weeks pregnant)	US	Endometriomas^∗^	Laparotomy (opening the cyst and stripping the lining)
Ueda et al. (2010) [2]	33	61	Second trimester	US	Ovarian endometriosis^∗^	Peritoneal washing and drainage
Reif et al. (2011) [10]	25	NA (after rupture)	Second trimester (27 weeks pregnant)	US	NA	Laparotomy (operative hemostasis and preterm cesarean section)
Present study	39	68 × 45 × 35	Second trimester (27 weeks pregnant)	US MRI	Rupture of decidualized endometriotic cyst	Conservative treatment, an elective cesarean section, and right ovarian cystectomy at 37-week pregnancy

NA: not applicable; US: ultrasonography; MRI: magnetic resonance imaging. ^∗^The preoperative diagnosis of the ruptured tumor was not described.
